# Trace Detection of Metalloporphyrin-Based Coordination Polymer Particles via Modified Surface-Enhanced Raman Scattering Assisted by Surface Metallization

**DOI:** 10.1155/2016/6394858

**Published:** 2016-12-26

**Authors:** Yu Sun, Alessio Caravella

**Affiliations:** ^1^International Institute for Carbon-Neutral Energy Research (WPI-I^2^CNER), Kyushu University, Motooka 744, Nishi Ku, Fukuoka 819-0395, Japan; ^2^Department of Environmental and Chemical Engineering, University of Calabria, Via Pietro Bucci, Cubo 44A, 87036 Rende, Italy

## Abstract

This study proposed a facile method to detect metalloporphyrin-based coordination polymer particles (Z-CPPs) in aqueous solution by modified surface-enhanced Raman scattering (SERS). The SERS-active particles are photodeposited on the surface of Z-CPPs, offering an enhanced Raman signal for the trace detection of Z-CPPs.

## 1. Introduction

Metalloporphyrin are compounds formed by a combination of porphyrin and metal ions, playing important roles in catalysis, fluorescence, sensing, optical imaging, electronics, photochemistry, and biological applications [1–6]. Recently, intensive investigation has been dedicated to morphology controlled synthesis of metalloporphyrin-based coordination polymer particles (CPPs) and several groups reported that diverse metalloporphyrin-based nanoscale/microscale particles can be synthesized through a bottom-up self-assembly process assisted by surfactants, such as Pluronic F127, sodium dodecyl sulfate (SDS), and hexadecyltrimethylammonium bromide (CTAB) [7–14]. Furthermore, it has been reported that metalloporphyrin-containing CPPs can be used for solar hydrogen generation and water purification in the aqueous solutions [13, 14]. Therefore, it is of significant importance to identify metalloporphyrin-based CPPs in the aqueous solution. In our previous works, a large amount of CPPs powder was required to identify CPPs through the X-ray diffraction (XRD) method, which usually requires complex processes, such as repeated centrifugation and sample drying at low temperature to obtain powder of samples for further identification [13, 14].

Metal-organic interactions have been the focus of intense multidisciplinary research in areas such as catalytic chemistry, materials science, and molecular electronics [15, 16]. Surface-enhanced Raman scattering (SERS) is widely recognized as a powerful tool for studying molecular adsorbates on metal surfaces and provides direct information on metal-molecule interactions, even in IR-opaque media such as aqueous solutions [17]. Typically, SERS is employed for (1) molecule detection in solutions or on substrates due to the molecular adsorption on SERS-active particles or SERS-active substrates [18, 19] and (2) molecule aggregates on SERS-active substrates [20]. Nevertheless, identification of self-assembled CPPs in the aqueous solution has been infrequently documented. To the authors' best knowledge, except for our idea proposed recently [21], no Raman data were obtained for CPPs in the solution even though coordination polymers have been intensively investigated. This is probably due to the low concentration of the analyte in the solution. Different from typical SERS, in which SERS-active materials, such as Au, Ag, and Cu particles, are usually employed as substrates for molecule adsorption or molecule aggregation, we employ the analyte, here CPPs, as substrate and attached SERS-active materials on their surface to enhance the detection signal in the aqueous solution.

## 2. Materials and Methods 

### 2.1. Materials

Zinc 5, 10, 15, 20-tetra(4-pyridyl)-21H, 23H-porphine (ZnTPyP), cetyltrimethylammonium bromide (CTAB), sodium tetrachloroaurate (III) dehydrate (NaAuCl_4_·2H_2_O), ascorbic acid from Aldrich Chemical Co., sodium hydroxide, and hydrochloric acid from Wako Chemicals were used without further purification. All solvents were prepared by using Milli-Q water.

### 2.2. Preparation of Stock Solutions

As ZnTPyP does not readily dissolve in water, its homogeneous stock solution (0.01 M) was prepared by dissolving an appropriate amount of ZnTPyP in HCl solution (0.2 M) to acidify its pyridyl groups, forming soluble tetrapyridium cations. The basic stock solution of surfactants was prepared by dissolving 0.01 M CTAB and 0.02 M sodium hydroxide in aqueous solution. The gold complex solution (10 mM) was prepared by dissolving NaAuCl_4_·2H_2_O in 10 mL aqueous solution. The electron donor solution (0.1 M) was freshly prepared by dissolving ascorbic acid in the aqueous solution.

### 2.3. Preparation of ZnTPyP-CPPs (Abbreviated as Z-CPPs)

An amount of 250 *μ*L of a ZnTPyP stock solution (0.01 M) was rapidly injected into 5 mL of a basic stock solution with vigorous stirring at room temperature.

### 2.4. Preparation of Au Decorated ZnTPyP-CPPs (Abbreviated as Au-Z-CPPs)

The solution of gold complexes (NaAuCl_4_·2H_2_O) together with freshly prepared ascorbic acid solution was added to the freshly prepared Z-CPPs solution. After mild stirring for 40 min under visible light illumination, the color of the mixture solution turned into light-reddish, indicating the successful photoreduction from gold complexes to gold nanoparticles. Raman spectra were recorded without further treatment (Figure 2).

### 2.5. Characterization

To prepare the samples for field-emission scanning electron microscopy (FE-SEM, TESCAN, and MIRA3) and transmittance electron microscopy, the as-prepared ZnTPyP particles were redispersed in pure water, dropped onto silicon wafer substrate and Cu grid, respectively, and finally dried at 50°C in the oven. The chemical state of gold element was determined with X-ray photoelectron spectroscopy (XPS, ESCA2000), which was performed with an Al K*α* and Mg K*α* X-ray source. Raman measurements were performed using a Renishaw 2000 Raman microscope system (Renishaw, UK). A Melles Griot HeNe Laser, operating at *λ* = 785 nm, was used as excitation source, with a laser power of approximately 15 mW. The Rayleigh line was removed from the collected Raman scattering using a holographic notch filter located in the collection path. The Raman scattering was collected using a charge-coupled device (CCD) camera at a spectral resolution of 4 cm^−1^. All spectra were calibrated to the 520 cm^−1^ silicon line. A 20x objective lens was used to focus a laser spot on the glass tube.

## 3. Results and Discussion

Briefly, zinc 5, 10, 15, 20-tetra(4-pyridyl)-21*H*, 23*H*-porphine (abbreviated to ZnTPyP) was chosen for the investigation and 250 *μ*L of a ZnTPyP stock solution (0.01 M) was rapidly injected into 5 mL of a basic stock solution at room temperature and under vigorous stirring. The solution turned cloudy immediately. However, in order to facilitate the growth of ZnTPyP-CPPs (abbreviated to Z-CPPs), the cloudy solution was kept at room temperature without stirring for 12 h. Then, we first measured the Raman spectrum of the as-synthesized Z-CPPs in the solution without any further treatment; however, no Raman signal was obtained.

**Scheme 1:**
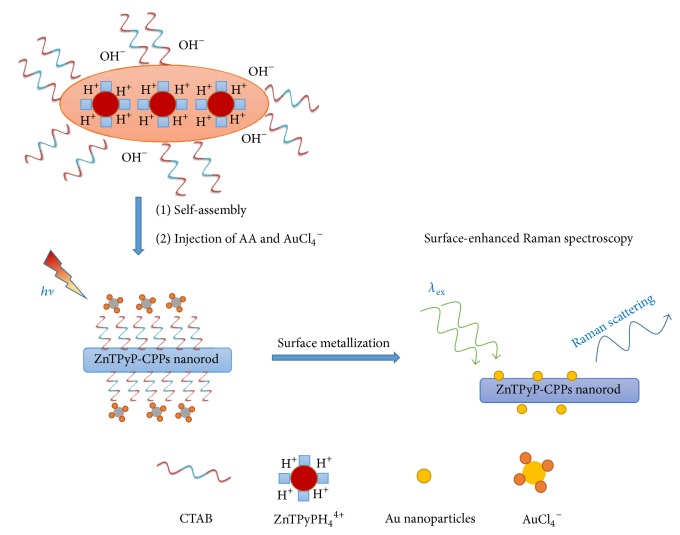
Proposed method for identification of Z-CPPs by SERS through surface metallization in the aqueous solution.

To design a method to detect Z-CPPs in the solution, we hypothesized that if SERS-active particles, such as Au particles, can attach on the surface of Z-CPPs, the trace detection could be possibly achieved in the aqueous solution. It was documented that the absorption of visible or UV-light by photocatalysts (PCs) yields the long-lived excited triplet *π*-*π* state, PCs^*∗*^, which is rapidly reduced by an electron donor (ED) such as ascorbic acid; if the long-lived radical anion has suitable electronic structures, it is capable of efficiently reducing a variety of metal ions including Ag, Au, Hg, Pb, Cu, and Pt to the zero-valent metals [10]. Therefore, as shown in Scheme 1, immediately after the synthesis of Z-CPPs, 0.5 mL of NaAuCl_4_·2H_2_O (10 mM) aqueous solution and 1 mL of ascorbic acid (0.1 M) aqueous solution were add to the Z-CPPs solution. The resulting mixture was gently stirred for 40 min under visible light illumination until the mixture turned into a light-reddish color.

**Figure 1:**
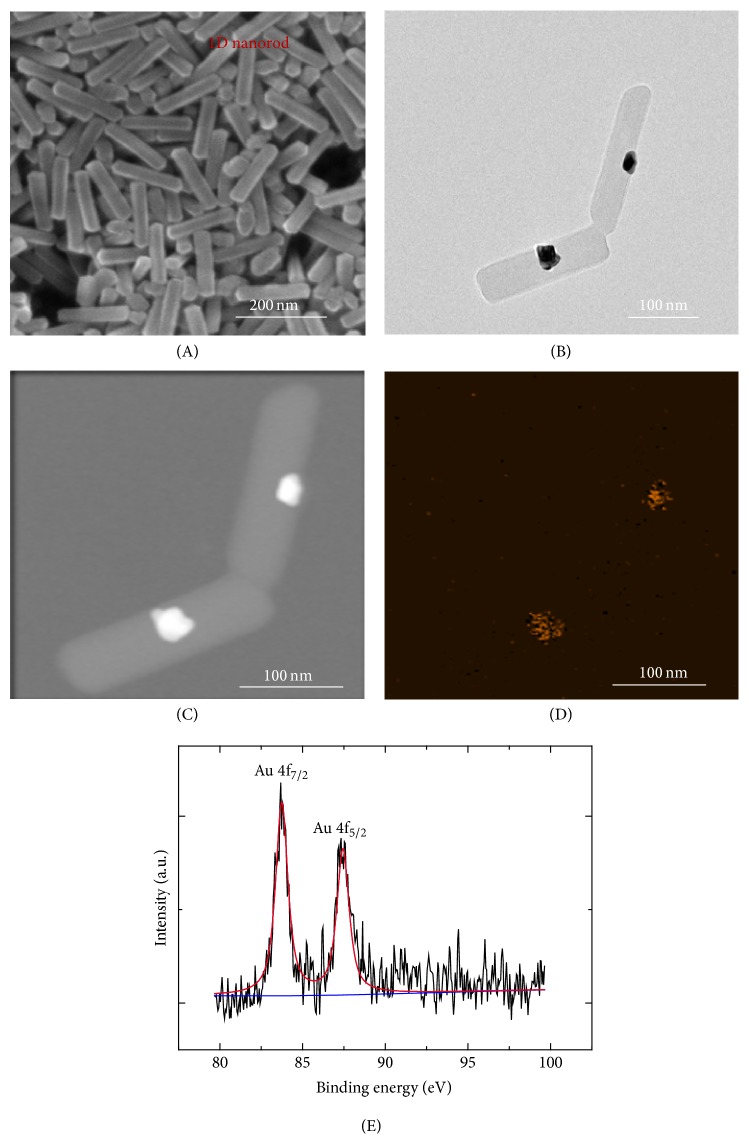
(A) SEM image of the Z-CPPs nanorod structures; TEM (B) and STEM (C) images of Au-Z-CPPs; elemental mapping data of (C) Au on ZnTPyP-CPPs; (E) XPS analysis of Au element for Au-Z-CPPs.

**Figure 2:**
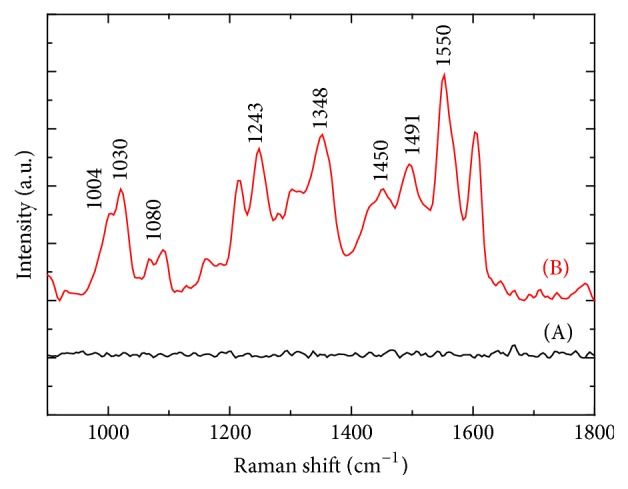
Raman spectra of (A) Z-CPPs and (B) Au-Z-CPPs in the aqueous solution.

The external morphologies of the synthesized Z-CPPs and Z-CPPs attached with gold particles (Au-Z-CPPs) were characterized by scanning electron microscopy (SEM) and transmittance electron microscopy (TEM). As shown in Figure 1(A), Z-CPPs nanorods with an average length of ~230 nm and width of ~80 nm were synthesized. The TEM image and STEM image (Figures 1(B) and 1(C)) of Au-Z-CPPs together with Au elemental mapping (Figure 1(D)) revealed that AuCl_4_^−^ complexes were reduced to Au nanoparticles under visible light illumination, which successfully attached on the surface of Z-CPPs. XPS analysis of Au element for collected powder samples (powder Au-Z-CPPs) is shown in Figure 1(E), which further confirmed that AuCl_4_^−^ were fully reduced to Au nanoparticles by observing XPS peaks at 83.8 eV and 87.3 eV, corresponding to Au 4f_7/2_ and Au 4f_5/2_ of metallic gold, respectively. The 1D Z-CPPs maintained their original structure after Au decoration.

**Figure 3:**
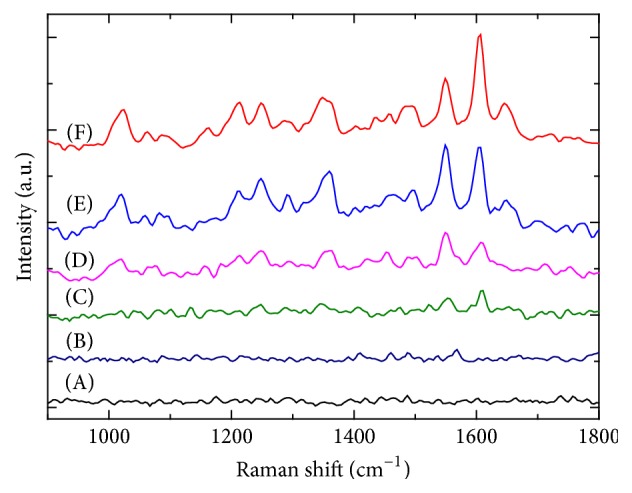
SERS spectra of Z-CPPs and Au-Z-CPPs according to the ZnTPyP molecule concentration varying from 500 to 10 nmol: (A) original Z-CPPs solution, Au-Z-CPPs with (B) 10 nmol, (C) 20 nmol, (D) 100 nmol, (E) 200 nmol, and (F) 500 nmol of ZnTPyP molecules.

As mentioned above, no Raman signal was obtained for Z-CPPs in the aqueous solution (Figure 3(A)). After Au decoration on the surface of the Z-CPPs nanorods, we carried out Raman spectroscopy measurement. It is illustrated that all characteristic group contributions (fingerprints) consistent with ZnTPyP structures were identified, including 1004 cm^−1^ [*ν*(C_*α*_–C_m_)], 1030 cm^−1^ [pyrr *δ*(C–H)], 1080 cm^−1^ [*δ*(C_*β*_–H)], 1243 cm^−1^ [*ν*(C_m_–Pyrr)] as well as the *φ* stretch normal modes, 1384 cm^−1^ [*ν*(C_*α*_–H)], 1450 cm^−1^ and 1491 cm^−1^ [*ν*(C_*α*_–C_*β*_)], and 1550 cm^−1^ [*ν*(C_*α*_–C_*β*_) and (C_*β*_–C_*β*_) stretch] [20]. This proved that our proposed method, SERS assisted by surface metallization on Z-CPPs (SM-SERS), is an effective approach for the detection of Z-CPPs in the aqueous solution.

In previous reports, we investigated morphology controlled synthesis of Z-CPPs and confirmed that the morphological transformation is related to solute concentration, temperature, and pH [13, 14]. Therefore, the investigation of the limit of detection for Z-CPPs through our SM-SERS method was carried out by diluting the Au-Z-CPPs aqueous solution in order to maintain its original structure. It is worth mentioning that dilution of the Au-Z-CPPs solution also causes a decrease in the concentration of active Au particles. Nevertheless, Figure 3 shows the SERS spectra of the Au-Z-CPPs aqueous solution corresponding to ZnTPyP molecule concentrations ranging from 500 to 10 nmol. As a reference, the SERS spectrum of the Z-CPPs aqueous solution is also shown in Figure 3(A). It was observed that the signal intensity decreased as the Z-CPPs concentration decreased and the limit detection was 10 nmol. This suggested that our SM-SERS method provides a high sensitivity for metalloporphyrin-CPPs detection. However, in determining the limit of detection, it was found that no SERS signal could be detected when the Z-CPPs concentration was lower than 10 nmol, which is probably due to the decreased concentration of active Au nanoparticles on the surface of the Z-CPPs.

## 4. Conclusion

In conclusion, we proposed the first demonstration to detect metalloporphyrin-containing CPPs in the aqueous solution through a designed SERS method (SM-SERS). Thanks to the photoactive properties of metalloporphyrins, we attached SERS-active gold nanoparticles on the surface of Z-CPPs via photoreduction of gold complexes under visible light illumination. Our proposed method was proven to be effective for Z-CPPs identification in that all characteristic group contributions (fingerprints) were consistent with ZnTPyP structures. The trace detection was achieved by obtaining the Raman spectra with a limit of detection for ZnTPyP of 10 nmol.
